# Nitrous oxide-induced myeloneuropathy: an emerging public health issue

**DOI:** 10.1007/s11845-022-02945-8

**Published:** 2022-02-12

**Authors:** John P. McCormick, Sophie Sharpe, Karen Crowley, Alexander Dudley, Ruadhan O’Laoi, Michael Barry, Lisa Owens, Colin P. Doherty, Janice Redmond, Sarah-Jane Yeung

**Affiliations:** 1 Department of Clinical Pharmacology and Therapeutics, St James’s Hospital, St James, Dublin, D08 NHY1 Ireland; 2 Department of Emergency Medicine, St James’s Hospital, St James, Dublin, D08 NHY1 Ireland; 3 Department of Neurology, St James’s Hospital, St James, Dublin, D08 NHY1 Ireland; 4 Department of Neurology, Beaumont Hospital, Dublin, Ireland; 5 Department of Endocrinology, St James’s Hospital, St James, Dublin, D08 NHY1 Ireland

**Keywords:** B12, Myeloneuropathy, Nitrous, Oxide

## Abstract

Increasing use of nitrous oxide as a recreational drug has been reported among young adults in western countries over the past decade. We present two cases of young males presenting to the Emergency Department (ED) of a large urban university hospital in Dublin with progressive neurological dysfunction related to nitrous oxide use. We review the pathophysiology, clinical features and treatment of nitrous oxide neurotoxicity. It is important that clinicians are aware of this evolving public health issue and are able to recognize the clinical features of this rare presentation, which may become more common in Irish EDs and GP surgeries as nitrous oxide abuse becomes more prevalent.

## Introduction

Increasing use of nitrous oxide as a recreational drug has been reported among young adults in western countries over the past decade [1–5]. Nitrous oxide is the fourteenth most popular recreational drug worldwide according to the most recent World Drug Survey [6].

While there is a paucity of data available regarding the prevalence of nitrous oxide use in Ireland, anecdotal reports from media outlets [7] and local drug surveillance centers [8] suggest that nitrous oxide use is increasing. Nitrous oxide canisters are readily available in Ireland and can be purchased through major online retailers, where they are marketed as “cream chargers.”

Cases documenting the adverse effects of nitrous oxide have been increasingly reported in the literature over the past decade. Here, we present two cases of young males presenting to the emergency department (ED) of a large urban university hospital in Dublin with progressive neurological dysfunction related to nitrous oxide use. We also review the pharmacological and clinical features of nitrous oxide neurotoxicity. It is important that clinicians are aware of this evolving public health issue and are able to recognize the clinical features of this rare presentation, which may become more common in Irish EDs and GP surgeries as nitrous oxide abuse grows.

### Case 1

A 20-year-old male presented to the ED with a 2-week history of progressive sensory changes affecting all four limbs and gait unsteadiness. He noticed mild numbness in his distal fingers and toes for several weeks, but this had begun to spread proximally with hyperalgesia and allodynia affecting the proximal upper and lower limbs. He became progressively unsteady on his feet in the days leading up to presentation.

The patient reported heavy nitrous oxide use twice a week for the preceding 6 weeks. He typically consumed twenty balloons per session but had recently stopped using individual canisters in favor of larger cylinders, which gave him a more intense effect. He was unable to accurately quantify his use as he often used a facemask directly connected to a cylinder. He reported daily cannabis use, as well as occasional cocaine and ketamine.

Clinical examination demonstrated a broad-based gait with a positive Romberg’s sign indicating a sensory ataxia as the cause of his unsteadiness. Sensory examination revealed loss of pin-prick sensation in the hands and feet to the wrists and knees bilaterally. Vibration sensation was reduced to the level of the ankles and wrists, with loss of proprioception sense in the fingers and great toes bilaterally. Power was mildly reduced distally, with 4/5 power observed in finger abduction and adduction, and ankle dorsiflexion. Tone and reflexes were normal in all limbs.

**Fig. 1:**
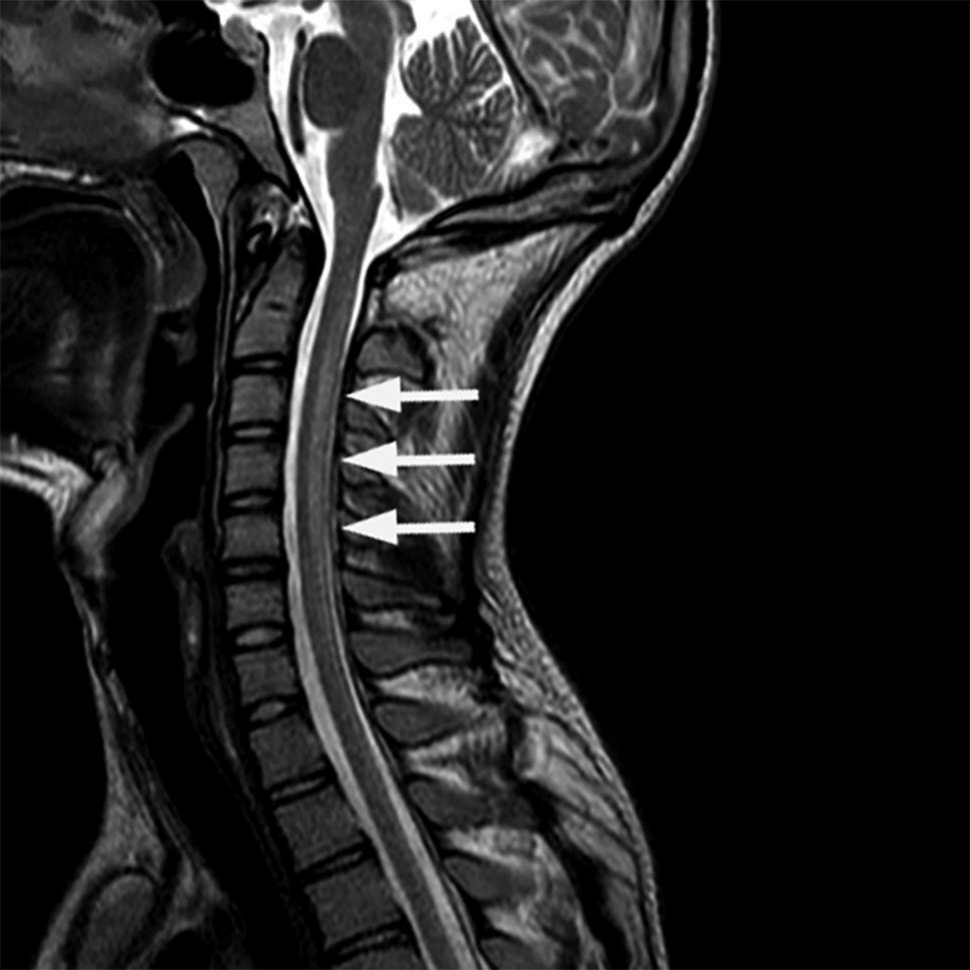
T2-weighted MRI; sagittal view showing increased signal in the dorsal columns from C2 to C7 (white arrows)

**Fig. 2:**
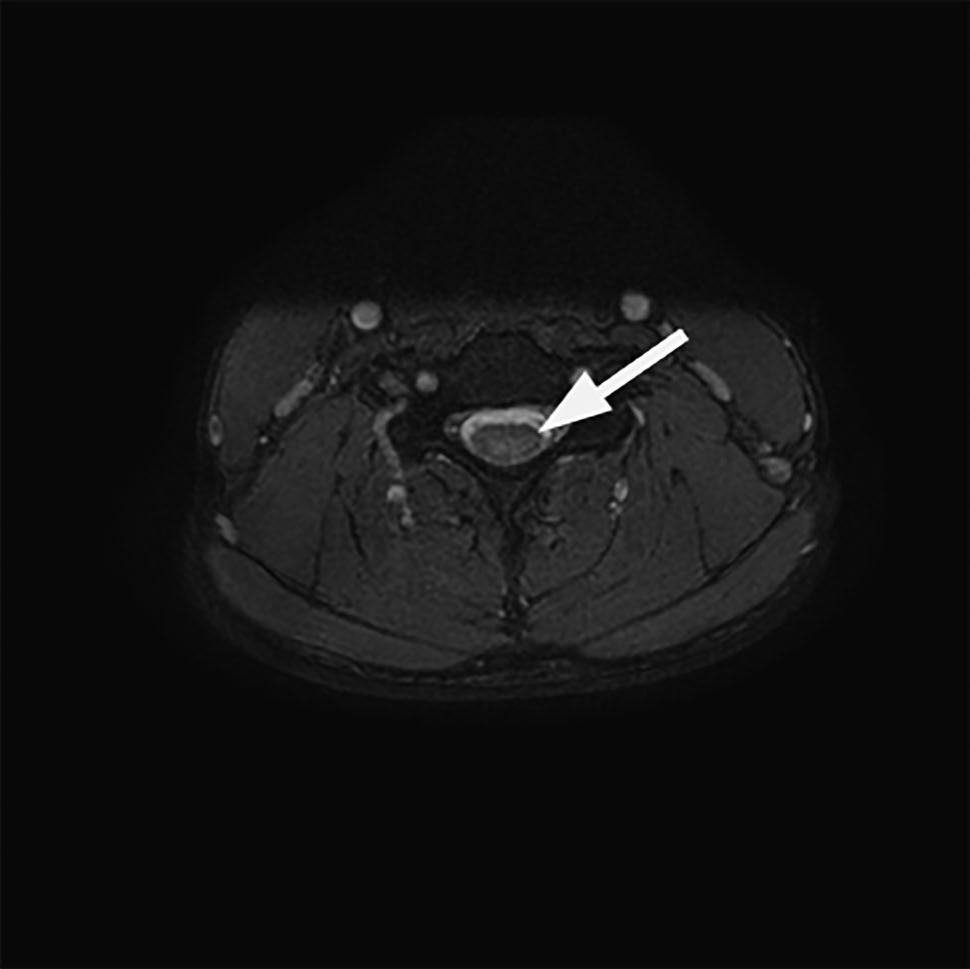
T2-weighted MRI; axial views showing increased signal in the dorsal columns at C5 (white arrow)

Initial laboratory investigations revealed a macrocytosis with low B12 and elevated serum homocysteine (Table 1). Magnetic resonance imaging (MRI) of the brain and spine (Figs. 1 and 2) revealed increased signal in the dorsal columns from C2 to C7. Nerve conduction studies (NCS) were poorly tolerated but were suggestive of motor axonal loss with no features of demyelination. The MR and electromyographic findings suggest a mixed central and peripheral sensory and motor neuropathy.

**Table 1:** Initial laboratory investigations

	**Patient 1**	**Patient 2**	**Reference range**
Serum			
Hb	13.9	15.3	(13.5–18.0 g/dL)
MCV	99.5	92.2	(83.0–99.0 fl)
WCC	7.4	9.9	(4.0–11.0 × 10^9^/L)
B12	155	271	(206–1,000 ng/L)
Folate	4.7	8.1	(4.5–20.0 μg/L)
Homocysteine	47.3	n/a	(4.0–12.0 μmol/L)
C-reactive protein	< 1	3.9	(0–5 mg/L)
Thyroid stimulating hormone	0.81	1.86	(0.27–4.20 mU/L)
CSF			
WCC	< 1	< 1	(0–5 WBC/cm^2^)
RCC	66—> 39	249—> 28	
Glucose	3.16	2.96	(2.22–3.89 mmol/L)
Protein	22	32	(15–45 mg/dL)
Culture	No growth	No growth	
Viral screen (enterovirus/ECHO/Coxsackie)	n/a	Negative

Intramuscular vitamin B12 and oral folic acid treatment were commenced in the ED and continued on alternate days for a planned duration of 2 weeks. Input from the multidisciplinary team included physiotherapy and occupational therapy. The patient discharged against medical advice after 7 days of treatment. During a virtual consultation 2 months post discharge, he reported improvement in his strength and balance, but significant residual paresthesia and numbness affecting his distal upper and lower limbs.

### Case 2

A 21-year-old male presented to the ED with a 2-week history of progressive tingling and weakness affecting all four limbs. The tingling spread proximally from his fingers and toes, and he developed weakness and impaired coordination when attempting fine motor tasks. He noticed that he was unable to distinguish keys from coins in his pockets and had fallen on the stairs several times in the days leading up to this presentation. As a construction worker, he worked on scaffolding and had taken time off work as he was unable to lift heavy objects. He stopped driving 3 days prior to his presentation as he felt he could not coordinate his limbs. His bowels had not opened in over a week, but he had not experienced any urinary symptoms.

The patient reported nitrous oxide abuse approximately once a fortnight over the preceding 7 months — usually between 10 and 40 balloons per session. Initially, he used small canisters of nitrous oxide, but recently had switched to larger cylinders of gas similar to oxygen cylinders, which were shared around at parties. His group of friends typically consumed five large cylinders of nitrous gas at a house party. He stopped using nitrous oxide 2 weeks prior to presentation. He drank approximately 20 units of alcohol per week and did not regularly use any other drugs.

On examination, the patient had an ataxic gait and was unable to walk unassisted. Proprioception was impaired in the upper and lower limbs with pseudoathetosis and a positive Romberg’s sign. Power was reduced in the small muscles of the hands but spared in the proximal muscles. Pin-prick, soft touch, and temperature sensation were significantly reduced in all limbs in a length-dependent pattern. Tone and reflexes were mildly reduced. Physical examination was otherwise unremarkable.

**Fig. 3:**
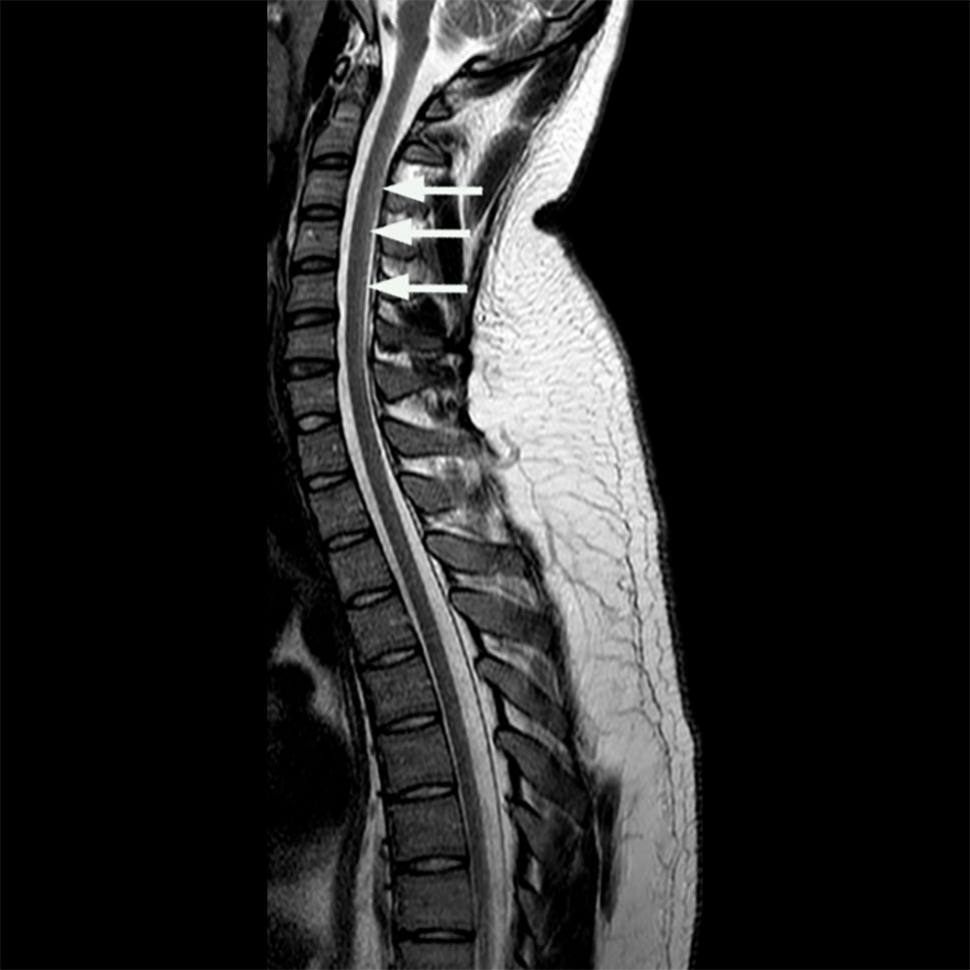
T2-weighted MRI; sagittal view of cervical and thoracic spine showing very subtle increased signal in mid and posterior cord (white arrows)

Initial laboratory investigations are shown in Table 1. Extended autoantibody, viral, and connective tissue disease screens were negative on both serum and cerebrospinal fluid (CSF). MRI of the brain and whole spine showed increased signal on T2-weighted imaging in the mid-posterior cervical and thoracic cord (Fig. 3). We did not have NCS in the second case but the MR findings and clinical examination suggest a mixed central and peripheral sensory and motor loss similar to the first case.

The patient was diagnosed with nitrous oxide-induced myeloneuropathy and treatment with high dose parenteral vitamin B12 and oral folic acid supplementation was commenced in the ED. His symptoms improved significantly over the following 10 days, and he regained the ability to walk independently. He was discharged home with neurology and neurorehabilitation outpatients reviews scheduled. At follow up 1 month post discharge, his motor symptoms had resolved sufficiently that he was able to walk unassisted, and he had returned to exercising in the gym. He reported minor persistent paresthesia and numbness in his distal fingers and toes.

## Pathophysiology

Nitrous oxide is a colorless, odorless, non-flammable gas which is commonly used as an anesthetic and analgesic agent in prehospital care, maternity services, and dentistry. It is also used as a fuel additive, an aerosol dispersant, and an additive to whipped cream in the catering industry. Nitrous oxide has become a popular recreational drug among young adults as it is easily available and has no significant “come down” or hangover effect after use. Its desired effects are short-lasting and may include a rush of dizziness, relaxation, laughing fits, auditory distortions, and sometimes hallucinations [1].

Most recreational users obtain nitrous oxide in the form of small silver canisters known as “whippets” or “silver bullets,” which are intended for use in catering. Users inhale the gas from balloons filled from these canisters. While most series suggest that typical recreational users consume less than 10 canisters per session, heavy use in excess of 700 canisters per day has been reported [9]. Nitrous oxide can also be obtained from larger cylinders intended for medical or industrial use. Inhalation of nitrous oxide via plastic bags or facemasks connected directly to these cylinders has been reported and is associated with higher rates of adverse events such as accidental asphyxiation or injury from falls [1, 3].

Following inhalation, nitrous oxide is rapidly absorbed via the pulmonary circulation. As a highly lipid soluble molecule, it readily crosses the blood–brain barrier, where it exerts its effects primarily via the opiate system; directly binding to mu, delta, and kappa opiate receptors [1, 2]. It may also work as an NMDA antagonist similar to ketamine [1, 3]. It has a rapid onset of action and is cleared from the body within hours. It is not detected by most commonly used drug testing kits, a feature which likely contributes to its popularity.

**Fig. 4:**
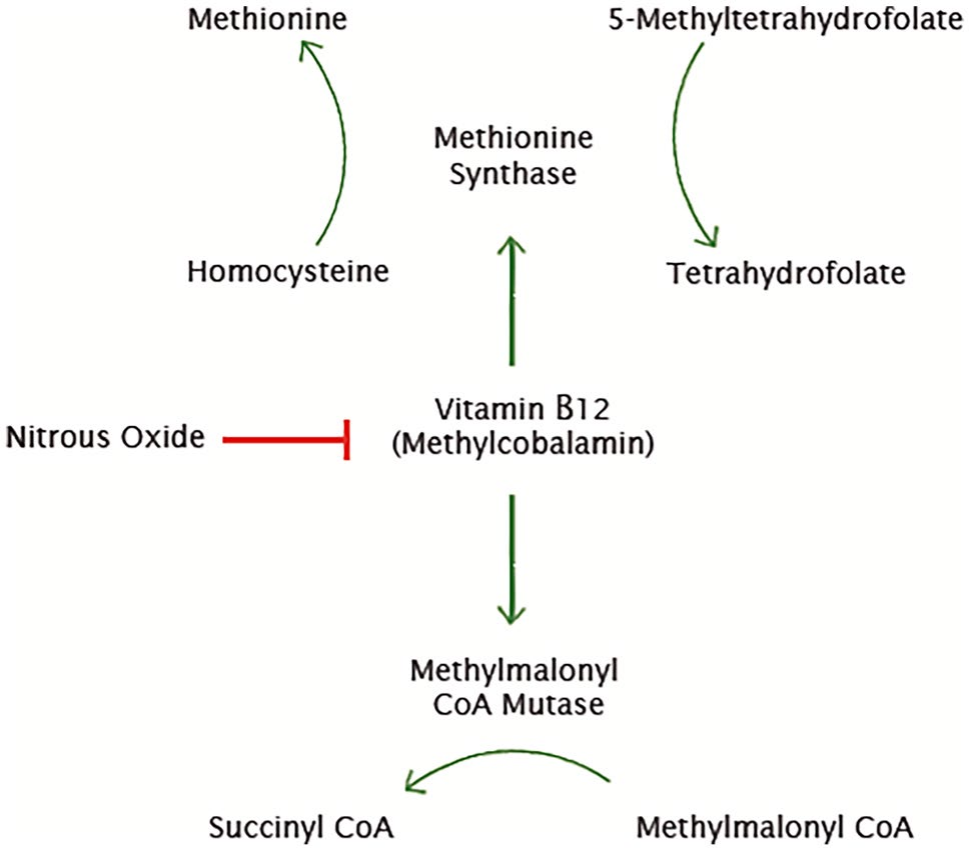
Flow chart showing pathways disrupted by the effect of nitrous oxide on B12

The neurotoxic effects of nitrous oxide are thought to be predominantly mediated by its effect on B12. It is postulated that nitrous oxide inactivates methylcobalamin by oxidizing the B12 cobalt ion from a 1 + to 3 + valence state [3]. This in turn interferes with numerous reactions in which B12 is a cofactor, including conversion of homocysteine to methionine; 5-methyltetrahydrofolate to tetrahydrofolate; and methyl-malonyl CoA to succinyl CoA (Fig. 4) [2, 3, 5, 9]. While the mechanisms by which inhibition of these reactions leads to myelin inflammation and neurotoxicity remain under debate, these observations form the basis of commonly used treatments including high dose B12 and methionine supplementation. They also explain the high serum homocysteine and methylmalonic acid levels which are commonly detected among patients with nitrous oxide-induced myeloneuropathy, even in the setting of normal B12 levels [3, 9].

Other B12-independent mechanisms of toxicity have been suggested including interference with the cytokine system [10], and NMDA antagonism leading to homocysteine imbalance [11, 12]. These hypotheses are supported by reported cases of progressive motor dysfunction despite appropriate supplementation of B12 and normalization of functional B12 marker levels [13].

## Clinical features

Low level neuropathic symptoms are common among nitrous oxide users, with 4.2% of recreational users reporting persistent paresthesia or numbness in one study [2]. The posterior columns are classically affected in more severe cases. Patients may describe poor balance and progressive difficulty with walking and fine motor tasks. Altered sensation is common, with distal numbness and paresthesia the most frequently reported initial symptoms [3]. Lhermitte’s sign may be present. As our cases illustrate, symptoms and signs can be highly variable with diffuse sensory changes, progressive motor weakness, and, in rare cases, autonomic dysfunction [9].

The differential diagnosis for such a presentation is broad and a thorough history and clinical examination including detailed drug history is crucial as patients may not volunteer their nitrous oxide use unless specifically asked.

Initial laboratory investigations where nitrous oxide toxicity is suspected should include a full blood count, serum B12 levels, protein electrophoresis, and CSF analysis including matched oligoclonal bands. Measured serum B12 levels and mean corpuscular volume may be normal in patients with nitrous oxide-induced myeloneuropathy and this does not exclude the diagnosis. Elevated serum homocysteine and methylmalonic acid levels help support the diagnosis but these tests may not be readily available outside large centers.

MRI brain and whole spine should be performed to exclude other pathologies and may show typical abnormalities such as V-shaped areas of high signal in the dorsal columns of the cervicothoracic cord on T2-weighted imaging [14]. The largest case series reported to date [9] found characteristic T2 hyperintensities in all twenty patients analyzed, while a 2016 systematic review of the case literature found positive neuroimaging reported in 39 out of 50 cases [3]. MRI changes may be subtle — in both of our cases, the abnormalities were only detected after multidisciplinary team review. Normal imaging does not exclude the diagnosis where there is a high clinical suspicion.

Nerve conduction studies may help support the diagnosis. A retrospective review of nerve conduction studies from 33 patients diagnosed with nitrous oxide-induced myeloneuropathy in Taiwan demonstrated significant motor and sensory amplitude reduction, conduction velocity slowing, and latency prolongation in the majority of patients when compared with healthy controls [15].

## Treatment and prognosis

The approach to treatment of nitrous oxide-induced myeloneuropathy is largely based on the case literature, and there are no major consensus guidelines available to guide the choice of treatment regimen or duration of therapy. Most patients will see significant improvement within months with appropriate treatment, although persistent sensory symptoms are not uncommon [3, 5, 9]. More severe residual impairment has been reported among patients who present late and those who continue to use nitrous oxide [5, 9].

Abstinence from further nitrous oxide consumption and parenteral B12 supplementation are the mainstay of treatment [1, 5, 9]. There is anecdotal evidence to suggest a role for methionine supplementation in cases where B12 alone is ineffective, although this may be limited by availability [16]. Some centers have reported use of folinic acid [17, 18], although this should only be used in addition to B12 supplementation to avoid exacerbation of functional B12 deficiency [19].

Our approach is to empirically administer intramuscular B12 to all patients in whom nitrous oxide-induced myeloneuropathy is suspected. We use 1,000 mg intramuscular hydroxocobalamin on alternate days for 2 weeks, followed by monthly doses thereafter until no further improvement in symptoms is seen. We also commence oral folic acid 5 mg once daily after initial B12 doses have been administered. A multidisciplinary team approach should be adopted to optimize outcomes, including input from physiotherapy and occupational therapy services with experience in neurorehabilitation.

## Abbreviations

ED: Emergency department
GP: General practitioner
MRI: Magnetic resonance imaging
